# The crosstalk between autophagy and ferroptosis in pulmonary fibrosis

**DOI:** 10.7555/JBR.39.20250243

**Published:** 2026-09-28

**Authors:** Rongzhu Liu, Dongnan Zheng, Fengxu Wang, Mengna Jiang, Rui Zhao, Shan Bao, Xinyuan Zhao, Demin Cheng

**Affiliations:** 1 Department of Occupational Medicine and Environmental Toxicology, Nantong Key Laboratory of Environmental Toxicology, School of Public Health, Nantong University, Nantong, Jiangsu 226019, China; 2 Department of Pulmonary and Critical Care Medicine, the First Affiliated Hospital of Soochow University, Suzhou, Jiangsu 215006, China; 3 Department of Respiratory, Wuxi Eighth People's Hospital, Wuxi, Jiangsu 214000, China; 4 China CDC Key Laboratory of Environment and Population Health, National Institute of Environmental Health, Chinese Center for Disease Control and Prevention, Beijing 100021, China

**Keywords:** pulmonary fibrosis, autophagy, ferroptosis, pathogenic mechanism, treatment strategies

## Abstract

Pulmonary fibrosis (PF) is a progressive lung disorder characterized by excessive deposition of extracellular matrix (ECM) in the alveoli, with irreversible fibrotic remodeling and destruction of alveolar structures. Clinically, PF is manifested by progressive dyspnea and a decline in lung function. These manifestations contribute to a poor prognosis and significantly diminish the quality of life of affected individuals. Current therapeutic options for PF remain limited, highlighting the urgent need to elucidate its molecular mechanisms in order to develop novel treatment strategies. Recent studies have revealed that autophagy, a lysosome-dependent cellular degradation process, and ferroptosis, an iron-dependent form of regulated cell death, engage in intricate crosstalk that may regulate PF pathogenesis. Autophagy maintains cellular homeostasis by mediating lysosome-dependent degradation, whereas ferroptosis is characterized by iron-dependent lipid peroxidation. The interplay between autophagy and ferroptosis plays a pivotal role in modulating fibrosis progression. However, the mechanistic interactions between autophagy and ferroptosis in PF remain poorly understood, particularly regarding their bidirectional crosstalk and their unique role in PF progression. This review aims to systematically synthesize the current understanding of autophagy-ferroptosis interactions in PF, thereby identifying potential therapeutic strategies and drug targets for PF treatment.

## Introduction

Pulmonary fibrosis (PF) is a lung disease in which excessive extracellular matrix (ECM) protein deposition leads to irreversible respiratory failure, tissue remodeling, and alveolar destruction^[1–2]^. Its pathology primarily involves recurrent alveolar epithelial injury, which triggers myofibroblast proliferation and differentiation and excessive ECM production^[3]^. Clinically, patients typically present with progressive dyspnea, dry cough, and a decline in lung function^[4]^. Idiopathic pulmonary fibrosis (IPF), a major form of PF, is characterized by interstitial fibrosis and progressive respiratory failure^[5]^. Median survival after diagnosis of IPF is only 3–5 years, and incidence and mortality rates are rising^[6]^. In 2014, the U.S. Food and Drug Administration approved pirfenidone and nintedanib for IPF treatment^[7]^. Although these antifibrotic agents partially inhibit ECM deposition, they cannot reverse established fibrosis.

During PF progression, epithelial impairment, notably alveolar type 1 cell injury, compromises the epithelial barrier. This triggers abnormal alveolar type 2 cell proliferation and differentiation, ultimately activating fibroblasts if left unresolved. M1 and M2 macrophages drive PF progression through distinct mediators, including inflammatory factors (*e.g.*, interleukin-1 [IL-1], inducible nitric oxide synthase, and IL-6) and fibrogenic agents (*e.g.*, IL-4, arginase-1, and IL-13). Fibroblast-to-myofibroblast transition (FMT), primarily driven by transforming growth factor-beta 1 (TGF-β1), results in ECM deposition and is central to PF pathogenesis. Endothelial cells constitute an additional myofibroblast source through endothelial-mesenchymal transition (EndoMT), adopting a myofibroblast-like phenotype characterized by high α-smooth muscle actin (α-SMA) and vimentin expression and low vascular endothelial cadherin expression^[8]^. Autophagy and ferroptosis critically regulate cellular pathophysiology during fibrosis initiation and progression^[9–10]^. The crosstalk between autophagy and ferroptosis is multifaceted. Autophagy may indirectly promote ferroptosis by dysregulating iron metabolism and impairing antioxidant defenses^[11]^. Conversely, ferroptotic lipid peroxidation products inhibit autophagic protection, thereby exacerbating cellular injury^[12]^. In the pathogenesis of IPF, autophagy and ferroptosis engage in complex crosstalk with cellular senescence and mitochondrial dysfunction^[13]^. This review comprehensively analyzes molecular interactions between autophagy and ferroptosis in PF pathogenesis and proposes potential targeted therapeutic strategies (***Fig. 1***).

**Figure 1:**
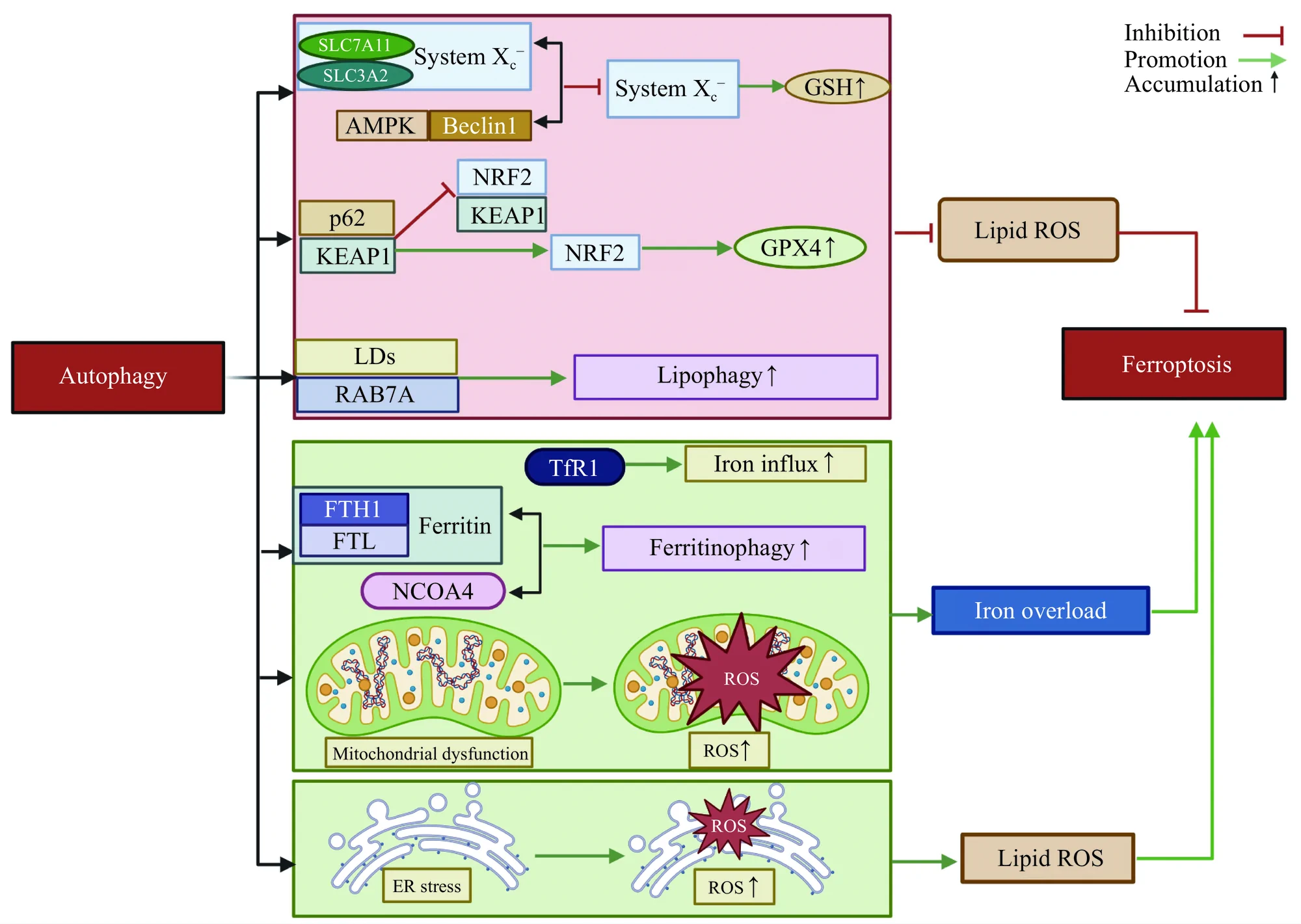
Schematic diagram of the crosstalk between autophagy and ferroptosis. The interaction between autophagy and ferroptosis involves multiple key pathways: (1) AMPK and Beclin1 suppress the activity of system X_c_^−^, resulting in a decrease in GSH levels and a subsequent reduction in antioxidant capacity. This cascade ultimately promotes the accumulation of lipid ROS and induces ferroptosis. (2) The interaction between p62 and KEAP1 prevents KEAP1-mediated inhibition of NRF2, facilitating NRF2 nuclear translocation. This process activates antioxidant genes, including GPX4. Increased GPX4 expression reduces lipid ROS levels, ultimately inhibiting ferroptosis. (3) Autophagy modulates lipid metabolism through RAB7A-mediated lipophagy. Enhanced lipophagic activity reduces lipid peroxidation, thereby suppressing ferroptosis. (4) Ferritin, composed of FTH1 and FTL subunits, undergoes autophagic degradation *via* NCOA4 binding (ferritinophagy), releasing free iron ions. Concurrently, TfR1-mediated iron uptake elevates intracellular iron levels. Iron overload promotes ROS accumulation and exacerbates lipid peroxidation, ultimately driving ferroptosis. (5) Mitochondrial dysfunction induces excessive ROS production, which damages cellular components and disrupts iron homeostasis, leading to iron overload. (6) Endoplasmic reticulum (ER) stress increases ROS generation, thereby promoting lipid peroxidation. Abbreviations: AMPK, AMP-activated protein kinase; ER, endoplasmic reticulum; FTH1, ferritin heavy chain 1; FTL, ferritin light chain; GPX4, glutathione peroxidase 4; GSH, glutathione; KEAP1, Kelch-like ECH-associated protein 1; LDs, lipid droplets; NCOA4, nuclear receptor coactivator 4; NRF2, nuclear factor erythroid 2-related factor 2; RAB7A, Ras-related protein Rab-7A; ROS, reactive oxygen species; SLC3A2, solute carrier family 3 member 2; SLC7A11, solute carrier family 7 member 11; system X_c_^−^, cystine/glutamate antiporter; TfR1, transferrin receptor 1.

## The mechanism of autophagy and ferroptosis

### The mechanism regulating autophagy

Autophagy is categorized into three types based on cargo delivery pathways and membrane dynamics: macroautophagy, microautophagy, and chaperone-mediated autophagy^[14–15]^. This review focuses on macroautophagy, which is the most extensively studied in PF. Macroautophagy, a complex cellular self-degradation process, involves multiple sequential steps^[16]^ as illustrated in ***Fig. 2***.

**Figure 2:**
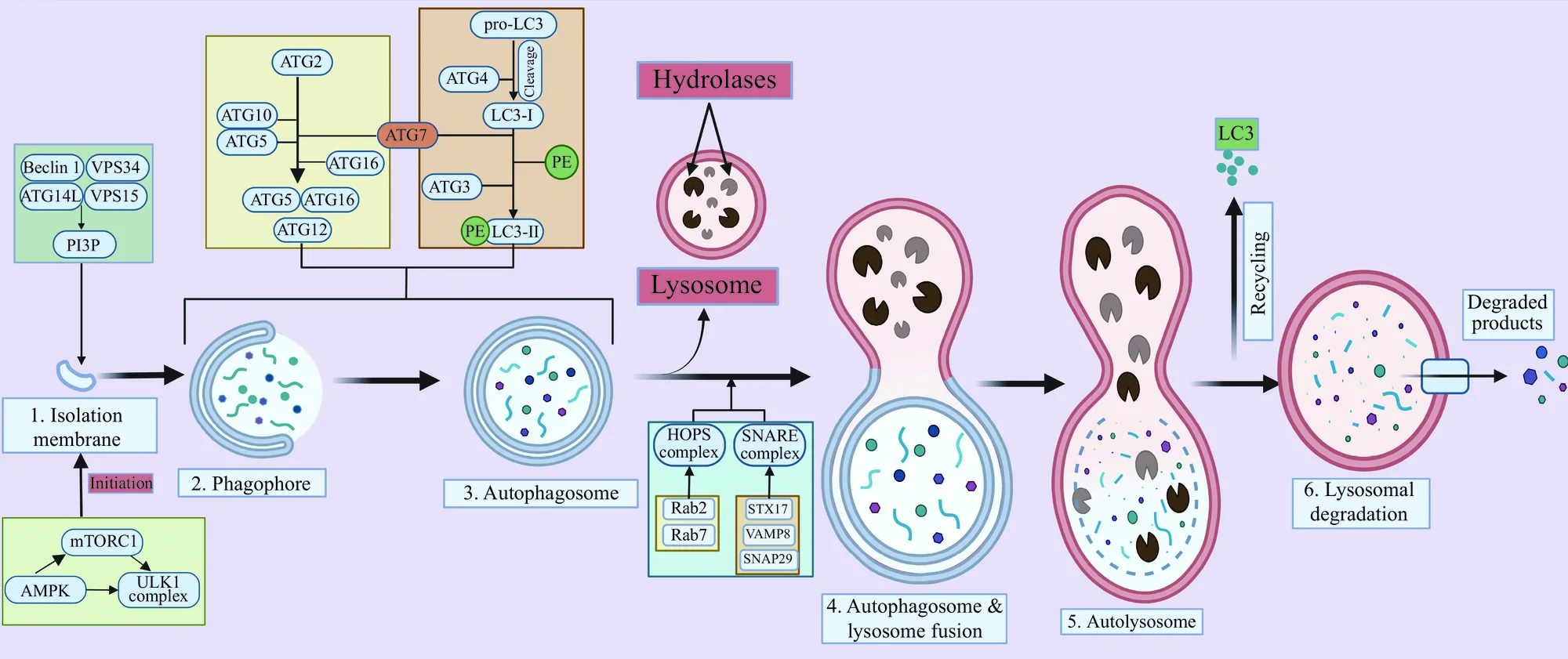
Molecular pathway of autophagy. Autophagy involves the following steps: (1) Initiation: Under energy stress, the ULK1 complex, regulated by mTOR/AMPK, is activated and translocates to the phagophore assembly site (PAS). (2) Nucleation and elongation: The Beclin1-VPS34 complex generates PI3P to promote membrane nucleation, recruits ATG proteins, and drives phagophore formation. (3) ATG complex formation: ATG12 combines with ATG5, then with ATG16L1, forming a complex that promotes phagophore membrane expansion and maturation. (4) LC3 lipidation: ATG4 cleaves pro-LC3 to generate LC3-Ⅰ, which combines with PE to form LC3-Ⅱ, inserting into the phagophore membrane to aid closure. (5) Autophagosome-lysosome fusion: The HOPS complex and SNARE proteins facilitate autophagosome-lysosome fusion, forming an autolysosome. (6) Degradation and recycling: Autolysosomal hydrolases break down macromolecules into basic units (*e.g.*, amino acids and fatty acids), which are released into the cytoplasm for reuse. Abbreviations: HOPS, homotypic fusion and vacuole protein sorting; LC3, microtubule-associated protein 1A/1B-light chain 3; LC3-Ⅰ, LC3 type Ⅰ; LC3-Ⅱ, LC3 type Ⅱ; mTORC1, mechanistic target of rapamycin complex 1; PE, phosphatidylethanolamine; PI3P, phosphatidylinositol 3-phosphate; pro-LC3, precursor form of LC3; Rab1, Ras-related protein in brain 1; Rab2, Ras-related protein in brain 2; SNAP29, synaptosomal-associated protein 29; SNARE, soluble N-ethylmaleimide-sensitive factor attachment protein receptor; STX17, syntaxin 17; ULK1, Unc-51-like autophagy-activating kinase 1; VAMP8, vesicle-associated membrane protein 8; VPS15, vacuolar protein sorting 15; VPS34, vacuolar protein sorting 34.

### The mechanism regulating ferroptosis

Ferroptosis is an iron-dependent form of cell death characterized by iron accumulation and lipid peroxidation^[17–19]^. The regulation of ferroptosis involves multiple critical steps (***Fig. 3***), which maintain cellular antioxidant defense homeostasis.

**Figure 3:**
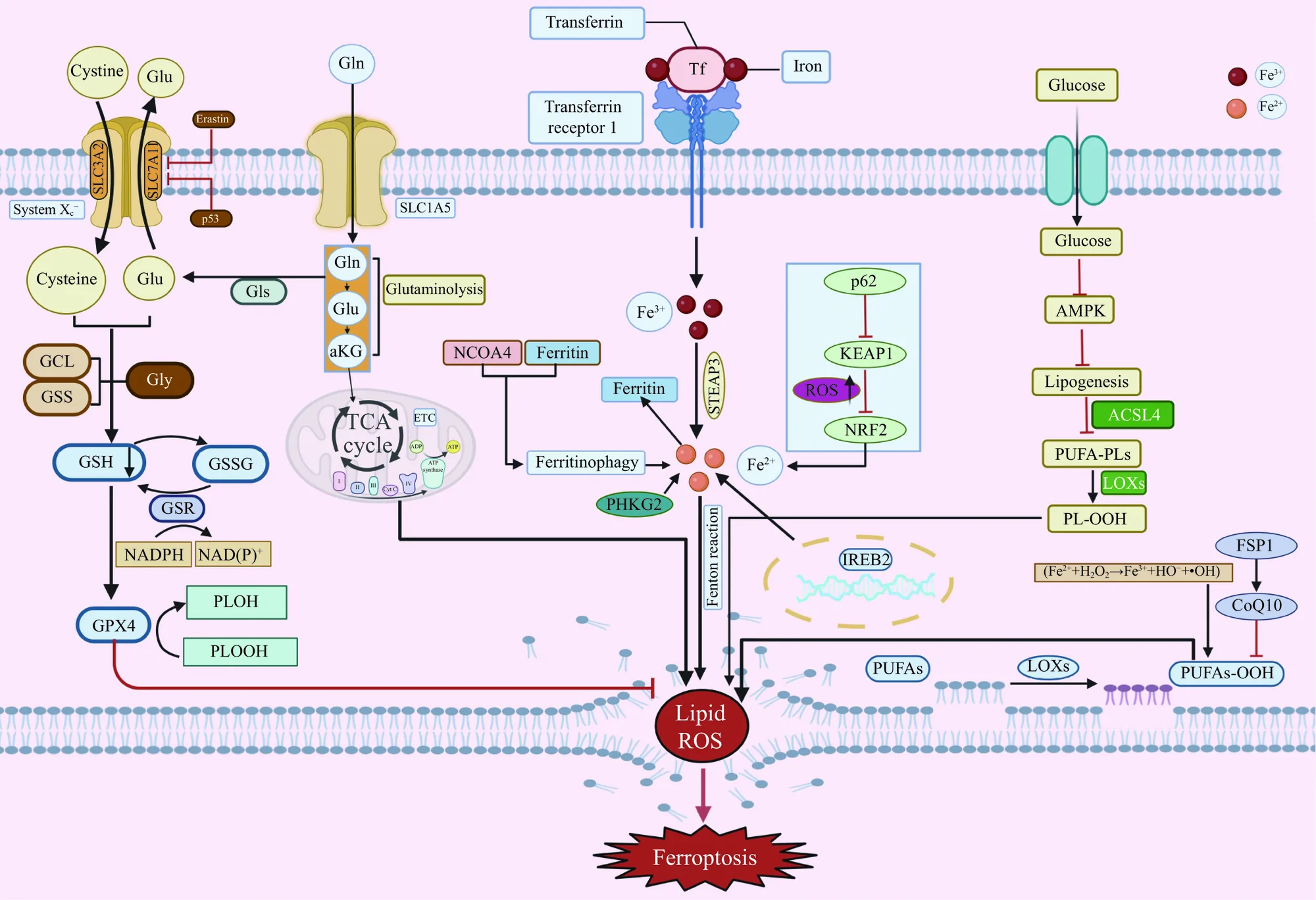
Molecular pathways of ferroptosis. Ferroptosis involves the following pathways: (1) System X_c_^−^ and GSH metabolism: System X_c_^−^ imports cystine to synthesize GSH, which is then utilized by GPX4 to counteract oxidative stress and inhibit ferroptosis. (2) Glutamine metabolism: Metabolites derived from glutamine enter the TCA cycle, leading to ROS production that promotes ferroptosis. (3) Iron metabolism: Cells take up Fe^3+^ and reduce it to Fe^2+^, which is stored in ferritin. NCOA4-mediated ferritinophagy releases Fe^2+^ to modulate ferroptosis. (4) Glucose metabolism: High glucose levels inhibit AMPK activity, thereby promoting *de novo* lipogenesis and sensitizing cells to ferroptosis. (5) Antioxidant defense: FSP1 and CoQ10 suppress lipid peroxidation and reduce ROS generation, thereby protecting cells from ferroptosis. (6) Mitochondria: As a key site for iron metabolism and ROS production, mitochondria are prone to oxidative damage, which in turn promotes ferroptosis. Abbreviations: ACSL4, acyl-CoA synthetase long-chain family member 4; CoQ10, coenzyme Q10; ETC, electron transport chain; FSP1, ferroptosis suppressor protein 1; GCL, gamma-glutamylcysteine ligase; Gln, glutamine; Glu, glutamate; Gly, glycine; GSS, glutathione synthetase; GSR, glutathione reductase; IREB2, iron-responsive element-binding protein 2; LOX, lipoxygenase; NAD(P)^+^, nicotinamide adenine dinucleotide (phosphate) (oxidized form); NADPH, nicotinamide adenine dinucleotide phosphate (reduced form); PHKG2, phosphorylase kinase gamma 2; PUFA-PLs, polyunsaturated fatty acid-phospholipids; SLC1A5, solute carrier family 1 member 5; STEAP3, six-transmembrane epithelial antigen of prostate 3; TCA cycle, tricarboxylic acid cycle; Tf, transferrin.

### The crosstalk mechanisms between autophagy and ferroptosis in PF

#### The crosstalk of signaling pathways

The mechanistic target of rapamycin (mTOR) signaling pathway plays a dual role in regulating both autophagy and ferroptosis^[20]^. As a central regulator of cellular metabolism, growth, and stress responses, mTOR functions through two primary complexes, mTOR complex 1 (mTORC1) and mTOR complex 2 (mTORC2), to modulate diverse cellular processes^[21]^. Under nutrient-rich and growth factor-abundant conditions, mTORC1 remains active and suppresses autophagy by phosphorylating the Unc-51 like autophagy activating kinase 1 (ULK1) complex. Conversely, during nutrient deprivation or cellular stress, mTORC1 activity is inhibited, relieving its suppression of the ULK1 complex and initiating autophagy. Additionally, mTORC1 inhibition also influences ferroptosis through multiple indirect mechanisms^[22]^. First, mTORC1 inhibition alters intracellular iron metabolism. For instance, autophagy activation redistributes intracellular iron and enhances its utilization efficiency, thereby modulating cellular susceptibility to ferroptosis^[23]^. Second, mTORC1 suppression downregulates the expression of antioxidant enzymes, such as glutathione peroxidase 4 (GPX4), a key regulator of ferroptosis. Reduced GPX4 levels significantly increase cellular vulnerability to ferroptosis^[24–25]^. Additionally, mTORC1 inhibition decreases the synthesis of monounsaturated fatty acids, diminishing the antioxidant capacity of cell membranes and further sensitizing cells to ferroptosis. Finally, mTORC1 inhibition activates autophagy, leading to increased ferritin degradation and the release of free iron ions, which further promotes ferroptosis. In summary, mTORC1 acts as a pivotal node in the interplay between autophagy and ferroptosis, influencing cellular fate through both direct and indirect mechanisms.

Beyond animal studies, evidence from human studies also suggests a role for the mTOR signaling pathway in PF. For instance, Lee *et al*^[26]^ found that mTOR expression was increased in epithelial cells of patients with PF. Moreover, the mTOR signaling pathway is activated in fibrotic areas of the lungs of PF patients^[27]^.

AMP-activated protein kinase (AMPK) plays a crucial role in regulating autophagy and ferroptosis under energy-deprived conditions in cells. The activation of AMPK inhibits mTORC1, thereby promoting autophagy, while also influencing ferroptosis by modulating iron metabolism and lipid peroxidation^[28]^. AMPK acts as a critical regulator of cellular energy status during autophagy^[29]^. Under energy stress, AMPK is activated and subsequently inhibits mTORC1 activity by phosphorylating tuberous sclerosis complex 2 (TSC2) or directly targeting mTORC1 components. The inhibition of mTORC1 alleviates its inhibitory effect on the ULK1 complex, initiating autophagy. As a central regulatory mechanism, AMPK activation orchestrates autophagy induction. In the context of ferroptosis, AMPK-mediated inhibition of mTORC1 enhances autophagy, which facilitates ferritin degradation and subsequent iron release, thereby promoting ferroptosis^[30]^. Additionally, AMPK modulates the expression of iron metabolism-related proteins, such as TfR1, indirectly affecting iron uptake and storage^[31]^. Moreover, AMPK interacts with mTORC1 and transcription factors, such as nuclear factor erythroid 2-related factor 2 (NRF2), affecting cell proliferation and differentiation, and thus influencing PF^[32]^. Therefore, it is essential to investigate the synergistic or antagonistic interactions of the AMPK pathway across various conditions. The schematic illustrating mTORC1, AMPK, and NRF2 signaling in both autophagy and ferroptosis regulation is shown in ***Fig. 4***.

**Figure 4:**
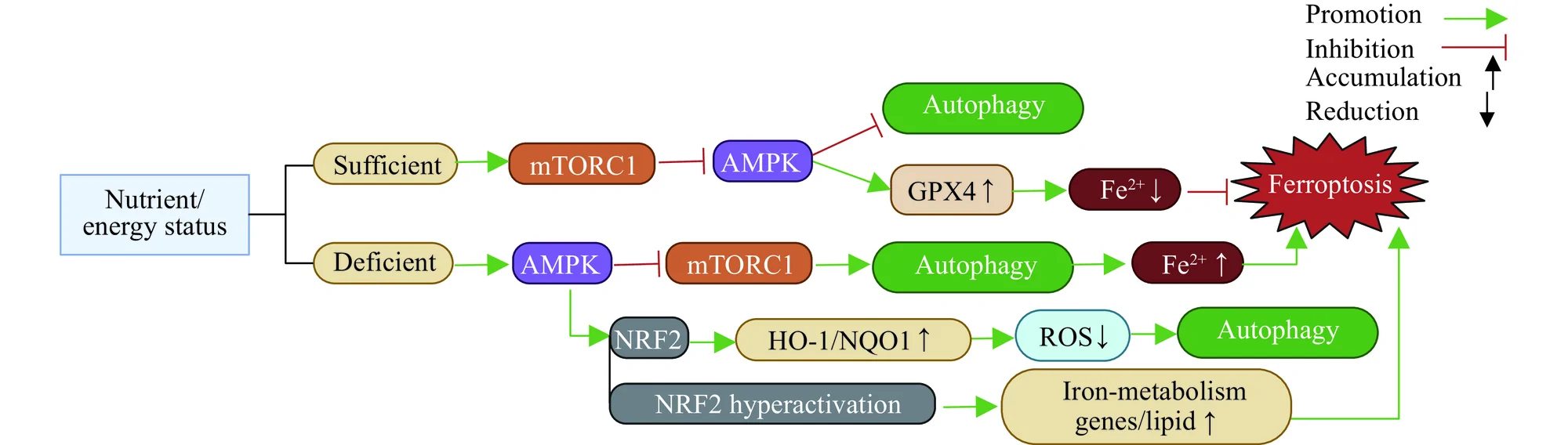
The crosstalk among mTORC1, AMPK, and NRF2 signaling pathways regulating autophagy and ferroptosis. Under energy-sufficient conditions (mTORC1-dominant), mTORC1 activation directly inhibits AMPK and autophagy while maintaining high GPX4 expression, thereby lowering Fe^2^^+^ levels and conferring ferroptosis resistance. Conversely, during energy deficiency or oxidative stress (AMPK-dominant), AMPK activation suppresses mTORC1 and triggers autophagy-mediated Fe^2^^+^ release; simultaneously, it phosphorylates NRF2 to initiate the antioxidant transcriptional program. NRF2 exhibits a dual regulatory role: its modest activation upregulates HO-1, NQO1, and other antioxidant genes, promoting ROS clearance and protective autophagy, whereas excessive activation under high-stress conditions induces iron-metabolism genes along with elevated lipid peroxidation, ultimately leading to ferroptosis. Abbreviations: AMPK, AMP-activated protein kinase; Fe^2+^, ferrous ion; GPX4, glutathione peroxidase 4; HO-1, heme oxygenase-1; NQO1, NAD(P)H quinone dehydrogenase 1; NRF2, nuclear factor erythroid 2-related factor 2; ROS, reactive oxygen species.

Currently, most basic science research on AMPK in PF focuses on cellular and animal models. The majority of existing studies indicate that the AMPK signaling pathway is inhibited in mouse models of PF^[33–34]^. Similar results were reported in mesenchymal stem cells (MSCs) derived from IPF patients, where the expression of p-AMPK was significantly decreased compared with control MSCs^[35]^.

NRF2 is a critical transcription factor that binds to antioxidant response elements to regulate the expression of several antioxidant molecules^[36]^. It plays a pivotal role in coordinating antioxidant defense, autophagy, and ferroptosis^[37]^. During autophagy, NRF2 influences the initiation and progression of autophagy by modulating the expression of autophagy-related genes. The activation of NRF2 signaling is mediated by the direct interaction between the NRF2 regulator Kelch-like ECH-associated protein 1 (KEAP1) and the adapter protein sequestosome 1/p62, which relieves KEAP1-mediated inhibition of NRF2 and facilitates NRF2 nuclear translocation. Additionally, NRF2 induces the expression of key autophagy factors, such as autophagy-related 5 (ATG5) and autophagy-related 7 (ATG7), thereby modulating autophagic activity^[38]^.

It is well known that there is a reciprocal relationship between autophagy and NRF2. Autophagy removes damaged or excessive mitochondria, thereby reducing ROS production. In parallel, selective autophagy can modulate NRF2 signaling through the p62/SQSTM1–KEAP1 axis. In turn, NRF2 upregulates the expression of antioxidant genes (*e.g.*, *HMOX1* and *NQO1*) and autophagy-related genes (*e.g.*, *P62*), mitigating lipid peroxidation and protecting cells from oxidative damage^[39]^. Notably, NRF2 activation also promotes autophagy, establishing a positive feedback loop crucial for cellular responses to oxidative stress^[40]^. The relationship between NRF2 and ferroptosis is complex. NRF2 regulates the expression of genes involved in iron metabolism, such as ferritin and iron transport proteins, thereby regulating intracellular iron levels^[41]^.

Current evidence primarily depends on cell and animal models, which limits clinical translation. Evidence from human studies supports a role for the NRF2 signaling pathway in PF, moving beyond findings from animal models. Wei *et al*^[42]^ reported that NRF2 expression was reduced in lung fibroblasts from patients with IPF. Furthermore, previous studies have demonstrated that patients with severe asthma, chronic obstructive pulmonary disease, or IPF exhibit deficient NRF2 activity^[43]^. Further research is required to explore its potential role as a therapeutic target.

#### Organelle interactions

Mitochondria are important organelles in autophagy and ferroptosis that play a crucial role in cellular function and fate^[44–45]^. Autophagy reduces ROS production by selectively clearing damaged mitochondria, thus inhibiting ferroptosis. Importantly, mitochondria are one of the primary sources of intracellular ROS^[46]^. Mitochondrial dysfunction and damage promote oxidative stress, often leading to excessive ROS production, which in turn induces ferroptosis. Under conditions of mild stress or during the initial phases of iron overload, autophagy reduces intracellular ROS accumulation by selectively removing these damaged mitochondria and thereby suppresses ferroptosis^[47]^. For example, GPX4 and ROS-damaged mitochondria are co-degraded through mitophagy, ultimately leading to hepatic ferroptosis^[48]^. Additionally, autophagy-mediated ROS reduction in mitochondria is a key mechanism for suppressing ferroptosis^[49]^. Mitochondria are not only a major source of ROS but also a critical site for intracellular iron metabolism^[50]^. Iron ion accumulation in mitochondria exacerbates ROS generation and ferroptosis. Mitochondria-specific ROS scavenging or ferroptosis inhibition can restore autophagy-dependent ferroptosis suppression, thereby alleviating excessive inflammation^[51]^. Dysregulated iron homeostasis mediates the crosstalk between ferritinophagy and mitophagy, which plays a critical role in PM2.5-induced ferroptosis and cardiac hypertrophy^[52]^. These mechanisms underscore the central role of mitochondria in regulating intracellular oxidative stress and iron metabolism, providing a theoretical foundation for developing therapeutic strategies for ferroptosis-related diseases.

Mitochondria are closely related to autophagy and ferroptosis in PF^[53]^. Iron accumulation in mitochondria increases ROS, promoting ferroptosis and accelerating PF. Most current studies are based on a single cell line and overlook mitochondrial functional variations across cell types. Future research needs to focus more on the role of mitochondria in patients with PF.

The ER is a complex, dynamic organelle responsible for protein folding, Ca^2+^ storage, and lipid and carbohydrate metabolism^[54]^. ER stress is a cellular stress response triggered by the accumulation of misfolded proteins, which induces activation of the unfolded protein response (UPR)^[55]^. If ER stress persists at a high level over an extended period, it can ultimately lead to cell apoptosis and ferroptosis^[56]^. For example, PM2.5 induces autophagic degradation of caveolin-1 through ER stress, thereby activating the TGF-β1/Smad3 axis to promote PF^[57]^. These stress responses further activate cellular senescence and apoptotic pathways.

The regulation of autophagy by ER stress is complex and dualistic. Under mild ER stress, autophagy is activated, facilitating the clearance of damaged mitochondria and proteins, reducing ROS, and thereby inhibiting ferroptosis. However, under severe ER stress, intracellular iron accumulation and ROS production increase significantly, leading to lipid peroxidation and ferroptosis^[58]^. In such cases, autophagy may be suppressed, impairing the clearance of damaged organelles. Regarding ferroptosis, ER stress and UPR activation can increase intracellular iron levels, promoting ferroptosis. Moreover, ER stress and mitochondria engage in extensive bidirectional crosstalk. This interplay, mediated by inflammatory cytokines, signaling pathways, and ROS, generates both positive and negative feedback loops that promote ferroptosis^[59]^. Furthermore, ER stress can regulate the expression of iron metabolism-related genes to drive ferroptosis^[60]^. It influences autophagy by regulating autophagy-related gene expression while also promoting ferroptosis by increasing ROS production. In this intricate biological process, autophagy and ferroptosis play crucial roles in maintaining intracellular homeostasis, offering potential therapeutic targets for PF.

ER stress plays a significant role in PF through its interactions with autophagy and ferroptosis. ER stress-induced autophagy may affect cell fate through its potential interactions with apoptosis and ferroptosis^[58]^. Severe ER stress leads to intracellular iron accumulation and a surge in ROS, triggering lipid peroxidation and ferroptosis^[61]^. The interplay between these two processes disrupts cellular homeostasis and exacerbates PF.

#### The crosstalk of key molecules

The key molecules involved in regulating autophagy and ferroptosis, along with their functions and targeted drugs, are summarized in ***Table 1***^[62–82]^.

**Table 1:** The key molecules involved in regulating autophagy and ferroptosis

Molecules	Functional mechanism in PF	Targeted drugs
NRF2	Inhibit the TGF-β1/Smad pathway, collagen deposition, antioxidant, and anti-inflammatory^[62]^	Activator: Rapamycin^[63]^, Tanshinone ⅡA^[64]^, Rosavin^[65]^
KEAP1	Promote NRF2 degradation and disrupt its imbalance, regulate cell proliferation and collagen deposition^[66]^	Inhibitor: Bardoxolone-methyl^[67]^, Sinomenine^[68]^
p62/SQSTM1	Facilitate autophagosome formation, regulate iron metabolism and TGF-β/Smad pathway^[69]^	Inhibitor: Bergenin^[70]^, Protodioscin^[71]^
mTORC1/AMPK	Regulate ECM production, involve the secretion of pro-inflammatory factors^[72]^	Inhibitor: Omipalisib^[73]^, Rapalogs^[74]^, Everolimus^[75]^
GPX4	Regulate autophagy and ferroptosis, block fibroblast activation and suppress EMT^[76]^	GPX4-inactivating agent: FINO2^[77]^, RSL3^[78]^
SLC7A11	Promote cystine uptake and GSH synthesis, thereby enhancing antioxidant capacity and suppressing ferroptosis^[79]^	Activator: Sulforaphane^[80]^ Inhibitor: Erastin^[81]^, Sorafenib^[82]^
Abbreviations: AMPK, AMP-activated protein kinase; ECM, extracellular matrix; EMT, epithelial-mesenchymal transition; GPX4, glutathione peroxidase 4; GSH, glutathione; KEAP1, Kelch-like ECH-associated protein 1; mTORC1, mechanistic target of rapamycin complex 1; NRF2, nuclear factor erythroid 2-related factor 2; p62/SQSTM1, sequestosome-1; PF, pulmonary fibrosis; ROS, reactive oxygen species; SLC7A11, solute carrier family 7 member 11; TGF-β1, transforming growth factor-beta 1.		

## The role of autophagy and ferroptosis in PF

### The role of autophagy in PF

Autophagy plays a complex and multifaceted role in PF, exhibiting both protective and pro-fibrotic effects. It is well established that PF involves multiple interacting mechanisms, including epithelial-mesenchymal transition (EMT), ECM deposition, myofibroblast differentiation, macrophage activation, and the secretion of inflammatory factors^[83]^. The protective role of autophagy is mediated by the degradation of ECM components (*e.g.*, collagen Ⅰ and Ⅲ)^[84]^, which reduces excessive deposition and ultimately mitigates fibrosis. Autophagy also inhibits EMT by degrading EMT-related transcription factors (*e.g.*, SNAI1 and SLUG), thereby slowing fibrosis progression^[85]^. Additionally, autophagy suppresses TGF-β-mediated myofibroblast differentiation, reduces the expression of fibronectin and α-SMA, and delays the progression of PF^[86]^. In the early stages of PF, autophagy maintains intracellular homeostasis by clearing damaged organelles and proteins, alleviating inflammatory responses, and inhibiting fibrosis progression^[87]^. However, under conditions of sustained injury and inflammation, impaired or excessive activation of autophagy may lead to pro-fibrotic effects^[88]^. For instance, downregulation of autophagy regulators such as Beclin 1 may exacerbate PF progression^[89]^. When autophagy is impaired or hyperactivated, it fails to clear excessive ECM, thereby promoting PF^[90]^. Autophagy deficiency may also intensify the differentiation of fibroblasts into myofibroblasts, further driving fibrosis^[91]^. Additionally, insufficient autophagy leads to the accumulation of damaged cellular components, driving cells into an irreversible senescent state and triggering the secretion of various inflammatory factors (*e.g.*, IL-6 and TNF-α) as well as growth factors (*e.g.*, TGF-β), which further promote inflammation and fibrosis^[92]^.

Lung fibroblasts from IPF patients show reduced autophagy. This autophagy deficiency impairs ECM degradation and promotes ECM deposition, thereby contributing to fibrosis^[93–94]^. This finding indicates that ECM synthesis is dysregulated in IPF fibroblasts, underscores the critical role of autophagy in this process, and suggests novel therapeutic avenues^[95]^. Autophagy has dual roles in PF. In the early stage, autophagy degrades excess ECM, suppresses EMT and myofibroblast differentiation, and clears damaged components, thereby reducing inflammation and maintaining homeostasis. However, under sustained injury or chronic inflammation, impaired or hyperactivated autophagy may paradoxically exacerbate fibrogenesis. Immune cell-mediated autophagy also plays an important role in the development of PF. A recent report found that macrophage Akt1 kinase-mediated mitophagy modulates apoptosis resistance and PF^[96]^. Additionally, suppressing 8-oxoguanine DNA glycosylase (OGG1) alleviates PF by blocking M2 macrophage polarization and subsequent mitophagy activation^[97]^. Current evidence relies heavily on animal models, and clinical validation remains limited. Future studies should investigate autophagy mechanisms in human patients to bridge translational gaps and improve therapeutic strategies.

Notably, autophagy-related genes identified in PF samples may hold potential for clinical diagnosis and targeted treatment of PF. For example, *MET* and *SH3BP4*, two autophagy-related genes identified from autophagy-associated differentially expressed genes between control and IPF samples (see GSE70866 dataset), effectively predicted IPF patient prognosis^[98]^. Moreover, a recent study found that increased LC3β accumulation in IPF lung tissue correlated with lower lung function values^[99]^.

### The role of ferroptosis in PF

The mechanism of ferroptosis in PF is complex, involving, among other aspects, iron metabolism, amino acid metabolism, lipid metabolism, and mitochondrial dysfunction. Iron ions generate hydroxyl radicals through the Fenton reaction, leading to lipid peroxidation and subsequent cell membrane damage^[100]^. A recent study demonstrated significantly elevated iron accumulation in PF patients, indicating that dysregulated iron metabolism and consequent ferroptosis may contribute to disease pathogenesis^[101]^. Although ferroptosis is not explicitly addressed in these studies, the findings implicitly suggest a critical role for iron accumulation in pulmonary fibrogenesis, with ferroptosis representing a potential mechanistic link between iron overload and fibrosis progression^[102]^. Iron accumulation may drive oxidative stress, amplify inflammatory responses, and disrupt cellular homeostasis, thereby promoting lung tissue damage and fibrotic remodeling. Future investigations should prioritize elucidating the mechanistic role of ferroptosis in PF and evaluating the therapeutic potential of inhibiting ferroptosis through modulation of iron metabolism and antioxidant systems, which may offer novel strategies for IPF treatment^[103]^.

The relationship between ferroptosis and amino acid metabolic disorders, particularly the depletion of glutathione (GSH) and GPX4, has been extensively investigated; however, whether these metabolic disorders directly induce ferroptosis remains controversial. On one hand, the depletion of GSH and GPX4 reduces cells' antioxidant capacity, leading to the accumulation of lipid peroxidation products and possible cell death^[104]^. On the other hand, the intricate nature of intracellular antioxidant systems implies that other compensatory mechanisms may play a significant role in various cell types and pathological conditions. Regarding therapeutic strategies, although the inhibition of GPX4 to induce ferroptosis in fibroblasts and attenuate collagen secretion is a novel approach, its associated risks warrant careful consideration. Such inhibition of GPX4 may trigger compensatory autophagy or pro-survival signaling pathways, potentially exacerbating fibrosis. This suggests that the regulation of ferroptosis is more complex than initially anticipated, exhibiting specificity that is related to cell type and disease stage^[105]^.

Mitochondrial dysfunction plays a critical role in ferroptosis. In PF, ferroptosis results in decreased mitochondrial membrane potential, mitochondrial DNA damage, and impaired mitophagy^[106]^. These alterations disrupt cellular energy metabolism and lead to excessive production of ROS, further promoting lipid peroxidation and cell death^[107]^. Subsequently, the release of iron ions and lipid peroxidation products activates inflammatory responses, promoting the infiltration of inflammatory cells and the release of inflammatory factors. Such inflammatory reactions exacerbate lung injury and further accelerate fibrosis progression^[108]^. Notably, the ferroptosis inhibitor ferrostatin-1 mitigates bleomycin-induced cell death, indicating that ferroptosis plays a critical role in bleomycin-induced lung injury^[109]^. Therefore, ferroptosis may contribute not only to direct cellular damage but also to PF progression by activating fibrosis-related pathways.

Dysregulated iron metabolism promotes ROS generation *via* the Fenton reaction, leading to lipid peroxidation and cellular damage that drive fibrogenesis^[110]^. This process depletes GSH and GPX4. However, whether GSH/GPX4 depletion directly causes ferroptosis remains controversial. Mitochondrial dysfunction, characterized by reduced membrane potential and DNA damage, disrupts energy metabolism and amplifies oxidative stress^[111]^. Ferroptosis releases iron ions and lipid peroxides, which activate inflammatory pathways, thereby worsening lung injury and accelerating fibrosis. Macrophage exosome-derived Ficolin B exacerbates bleomycin-induced PF through ferroptosis mediated by the cyclic GMP-AMP synthase-stimulator of interferon genes (cGAS-STING) signaling axis^[112]^. Furthermore, ferroptosis in dendritic cells promotes silica-induced lung inflammation and fibrosis by activating the retinoic acid signaling pathway^[113]^.

Notably, ferroptosis-related genes in PF samples may serve as potential biomarkers for the clinical diagnosis and treatment of PF. Yue *et al*^[114]^ performed a bioinformatics analysis and identified *ATM*, which encodes a key serine/threonine kinase, as a co-expressed hub ferroptosis biomarker gene in both the pulmonary tissues and peripheral blood of IPF patients. Additionally, Li *et al*^[115]^ found that ferroptosis-related genes (*ACO1*, *NRAS*, *ENPP2*, *MUC1*, and *ZFP36*) in bronchoalveolar lavage fluid could serve as prognostic biomarkers for IPF.

### The crosstalk between ferroptosis and autophagy in PF

The dual role of autophagy in ferroptosis has been extensively studied; however, existing research still has limitations. Autophagy promotes ferroptosis through various mechanisms, including ferritin degradation, TfR1 upregulation, and the degradation of lipid droplets and damaged mitochondria. These processes release iron ions and ROS, thereby driving the progression of ferroptosis. For example, NCOA4-mediated ferritinophagy degrades ferritin in lysosomes, releasing Fe^2+^ and exacerbating lipid peroxidation and ROS production^[116]^. These mechanisms have been demonstrated to occur in autophagy-dependent ferroptosis, underscoring the crucial role of autophagy. However, this complexity is often oversimplified, as most studies ignore the variations between cell types and even tissue types. Macrophages secrete IL-4 to regulate the sensitivity of epithelial cells to ferroptosis; however, the specific mechanisms underlying these cell-to-cell interactions remain unclear^[117]^. Future research should explore the synergistic mechanisms of autophagy and ferroptosis within a multicellular interaction network to gain a more comprehensive understanding of their pathological and physiological roles in PF.

During ferroptosis, excessive ROS accumulation can induce autophagy through the AMPK and NRF2 signaling pathways^[118]^. Additionally, iron ions can modulate autophagic activity indirectly by regulating iron metabolism-related proteins such as *NCOA4* and *TFR1*. Nuclear receptor coactivator 4 (NCOA4) mediates ferritinophagy, which involves the degradation of ferritin and the subsequent release of iron ions^[119–120]^. Under iron-replete conditions, NCOA4 binds to HERC2 and undergoes degradation, thereby inhibiting ferritinophagy. *NCOA4*-knockout mice show increased ferritin accumulation, reduced autophagic activity, and impaired iron availability^[121]^. Furthermore, increased iron induces toxicity by generating hydroxyl radicals *via* the Fenton reaction. These radicals mediate the oxidation of lipids, proteins, and DNA, thereby inducing autophagy and influencing PF progression^[122]^. However, whether such damage sufficiently triggers comprehensive autophagic activation and the precise mechanistic role of autophagy in ferroptosis remains to be further explored.

Recent studies have shown that autophagy, a key process for maintaining cell balance and metabolism, strongly interacts with ferroptosis. In fibroblasts, autophagy-mediated ferritin breakdown induces ferroptosis. This suggests that under certain conditions, ferroptosis may be a form of cell death that depends on autophagy. The ferroptosis inducer erastin stimulates enhanced autophagic activity, thereby promoting ferroptosis through iron accumulation and lipid oxidation. The interplay between autophagy and ferroptosis is critically mediated by ROS-dependent mechanisms^[123]^. As key cellular signaling molecules, ROS trigger autophagic responses under stress conditions, which facilitate autophagic degradation of ferritin and consequently increase intracellular labile iron pools. Subsequently, this elevated iron amplifies oxidative stress through enhanced lipid peroxidation, ultimately promoting ferroptosis. Furthermore, activation of the AMPK/mTOR signaling pathway in AECs promotes autophagy-mediated ferroptosis, which exacerbates PF^[124]^. Targeting ferroptosis and autophagy through modulation of the Sesn2/AMPK/NRF2 signaling pathway may mitigate bleomycin-induced PF^[125]^. Therefore, the intricate interplay between autophagy and ferroptosis presents promising avenues for developing novel therapeutic strategies targeting diseases associated with disrupted iron metabolism, such as PF.

In summary, this dynamic crosstalk between autophagy and ferroptosis in PF underscores the clinical imperative to precisely regulate autophagy and iron metabolism to disrupt pathological feedback loops and mitigate fibrotic progression (***Fig. 5***). However, current research is limited by oversimplified experimental models, unresolved molecular-level paradoxes, and limited therapeutic strategies. Future research must integrate multidisciplinary innovations, including spatial transcriptomics, real-time dynamic imaging, and clinical-translational methodologies, to achieve precision-targeted interventions for PF.

**Figure 5:**
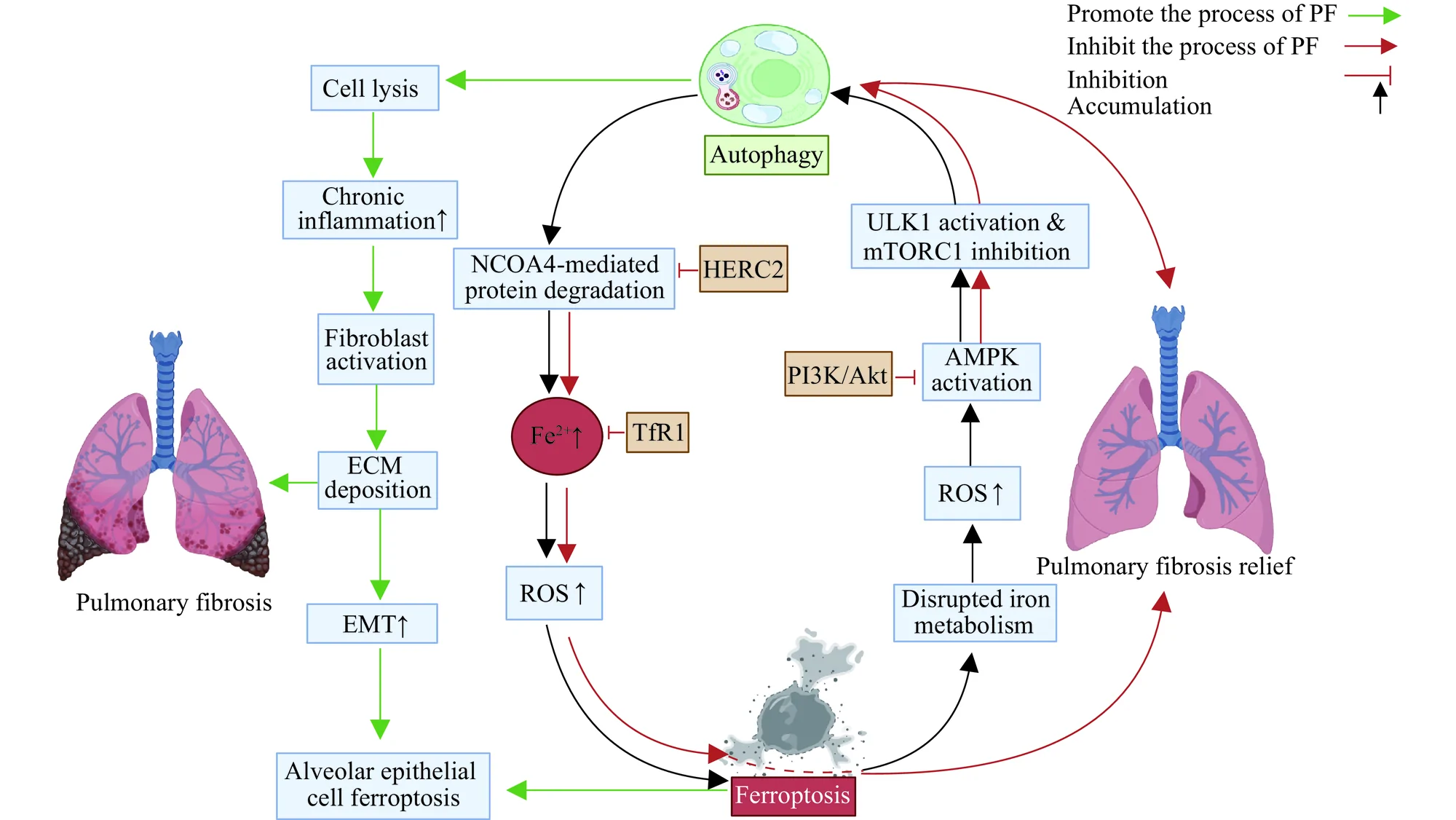
The dynamic crosstalk between autophagy and ferroptosis in pulmonary fibrosis (PF). The crosstalk between autophagy and ferroptosis in PF involves a feed-forward cycle where autophagy promotes ferroptosis through iron accumulation, and ferroptosis in turn enhances autophagy *via* AMPK signaling. This synergistic interaction drives PF through EMT, fibroblast activation, and excessive ECM deposition. Specifically, (1) Autophagy activates NCOA4-mediated protein degradation, increasing Fe^2+^, which in turn increases ROS, leading to ferroptosis; (2) Ferroptosis causes disrupted iron metabolism, increasing ROS, activating AMPK, promoting ULK1 activation and mTORC1 inhibition, further activating autophagy, forming a cycle; (3) Under specific pathological conditions, dysregulated autophagy may contribute to PF by promoting cellular injury and chronic inflammation, thereby facilitating fibroblast activation and ECM deposition; (4) Ferroptosis damages alveolar epithelial cells, leading to excessive extracellular matrix deposition, ultimately resulting in pulmonary fibrosis; (5) HERC2 inhibits protein degradation mediated by NCOA4, while TfR1 directly reduces iron ion levels; both decrease ROS production, suppress ferroptosis, and promote anti-fibrotic effects; (6) The PI3K/Akt pathway inhibits the activation of AMPK, indirectly modulating ULK1 and mTORC1, thereby affecting autophagy, which contributes to anti-fibrotic effects. Abbreviations: AMPK, AMP-activated protein kinase; ECM, extracellular matrix; EMT, epithelial-mesenchymal transition; HERC2, HECT and RLD domain-containing E3 ubiquitin protein ligase 2; mTORC1, mechanistic target of rapamycin complex 1; NCOA4, nuclear receptor coactivator 4; PI3K/Akt, phosphoinositide-3 kinase/protein kinase B; ROS, reactive oxygen species; TfR1, transferrin receptor 1; ULK1, Unc-51 like autophagy activating kinase 1.

### Therapeutic opportunities targeting ferroptosis and autophagy

Collectively, impaired autophagy flux in AECs may lead to abnormal protein accumulation and mitochondrial dysfunction, which in turn induces ER stress and the release of profibrotic factors. Furthermore, autophagy dysregulation in fibroblasts promotes fibroblast activation and ECM deposition. Evidence from human IPF tissue samples indicates significantly increased expression of autophagy-related protein markers relative to non-IPF lungs^[99]^. As such, autophagy-targeted therapeutic strategies and drugs hold promising potential for PF treatment.

Concurrently, ferroptosis plays an essential role in PF progression, and ferroptosis in AECs may be an early event in PF pathogenesis. Profibrotic cytokines released by AECs undergoing ferroptosis promote the transformation of fibroblasts into myofibroblasts. Evidence from human IPF samples reveals high expression of ferroptosis-related genes, which correlates with poor prognosis^[126]^. Therefore, targeting ferroptosis represents a novel, potential antifibrotic therapeutic approach for PF management.

In conclusion, targeting autophagy and ferroptosis represents a potential therapeutic strategy for PF. Several experimental agents, including deferoxamine, ferrostatin-1, liproxstatin-1, and berberine, have shown antifibrotic effects in preclinical studies, whereas nintedanib and pirfenidone are clinically established antifibrotic therapies that may also intersect with pathways related to autophagy and ferroptosis^[127]^. Potential alternative therapeutic drugs based on the molecular characteristics associated with autophagy and ferroptosis are listed in ***Table 2***. Additionally, the graphical summary of therapeutic agents targeting autophagy and ferroptosis is shown in ***Fig. 6***.

**Table 2:** Potential alternative therapeutic drugs against PF

Drugs	Mechanism	Evidence stage	Ref.
Deferoxamine (iron chelator)	By chelating intracellular iron, deferoxamine suppresses iron-dependent lipid peroxidation and inhibits ferroptosis, thereby attenuating PF progression.	Preclinical	[128–129]
Ferrostatin-1 (ferroptosis inhibitor)	By inhibiting ferroptosis through ROS scavenging, ferrostatin-1 reduces lipid peroxidation and prevents iron-dependent cell death, thereby attenuating ECM deposition and PF.	Preclinical	[130–131]
Liproxstatin-1 (ferroptosis inhibitor)	By inhibiting ferroptosis through suppression of lipid peroxidation, liproxstatin-1 reduces inflammation and ECM deposition, thereby alleviating PF.	Preclinical	[132–133]
Nintedanib	Through multi-target inhibition of PDGFR/FGFR/VEGFR and disruption of TGF-β/SMAD3 signaling, nintedanib reduces fibroblast activation, ECM deposition, and collagen production.	Specific clinical trial for PF	[134–135]
Pirfenidone	Pirfenidone combats PF through TGF-β1/Smad3 inhibition, antioxidant activity, anti-inflammatory activity, and suppression of ECM deposition, collectively suppressing PF.	Specific clinical trial for PF	[136–137]
Metformin (AMPK activator)	Metformin activates AMPK to suppress myofibroblast fibrogenic activity and inhibits the TGF-β1 signaling pathway, collectively attenuating PF.	Clinical investigation	[138–139]
Rapamycin (mTORC1 inhibitor)	Rapamycin attenuates PF by inhibiting mTOR to suppress cell proliferation, and blocking TGF-β/Smad signaling and EMT, synergistically reducing ECM synthesis.	Preclinical	[140–141]
Berberine (autophagy activator)	Through dual activation of autophagy and suppression of ferroptosis, complemented by antioxidant and anti-inflammatory effects, Berberine reduces ECM deposition to alleviate PF.	Preclinical	[142–143]
Abbreviations: FGFR, fibroblast growth factor receptor; PDGFR, platelet-derived growth factor receptor; ROS, reactive oxygen species; VEGFR, vascular endothelial growth factor receptor.			

**Figure 6:**
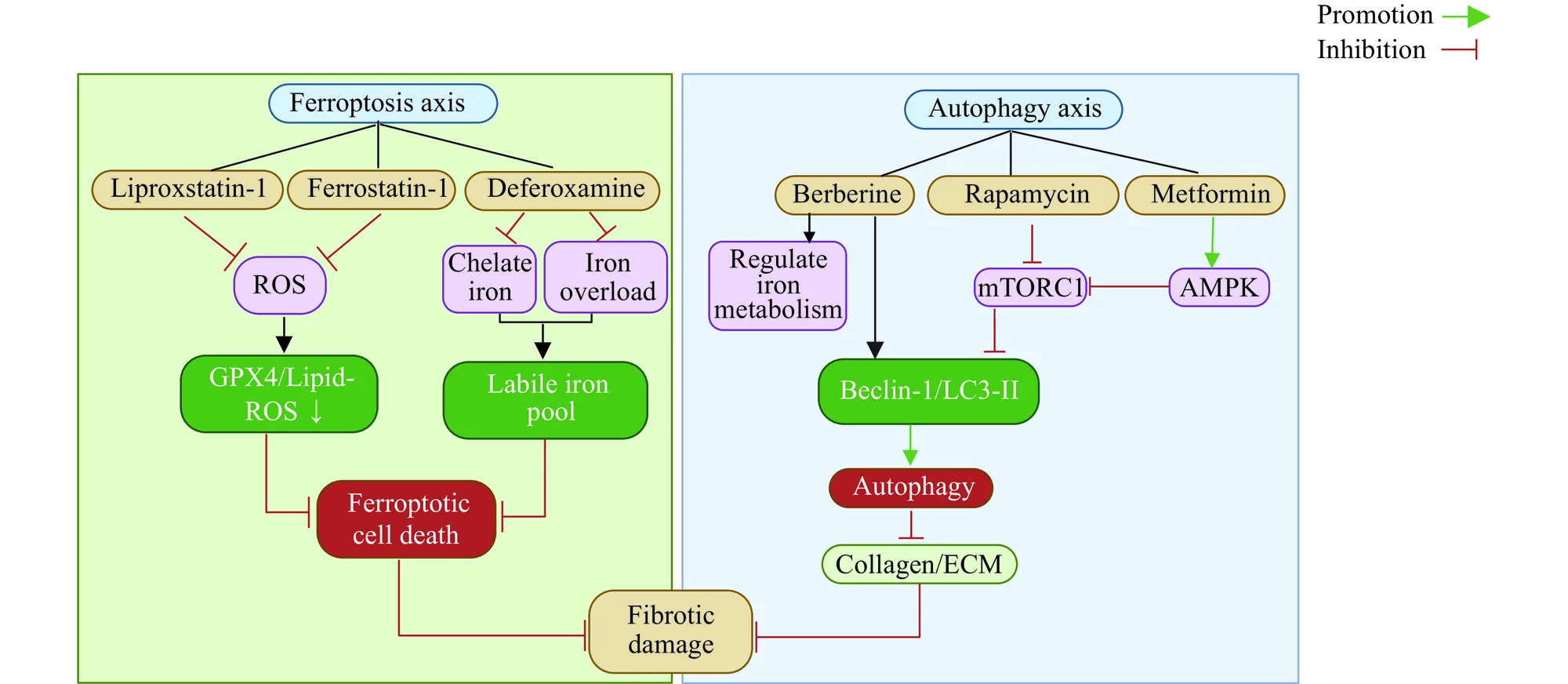
The graphical summary of therapeutic agents targeting ferroptosis and autophagy. This schematic illustrates the therapeutic targeting of ferroptosis and autophagy in pulmonary fibrosis. The framework links iron-dependent lipid peroxidation, autophagic dysregulation, and ferroptosis to fibrotic progression. Abbreviations: AMPK, AMP-activated protein kinase; ECM, extracellular matrix; GPX4, glutathione peroxidase 4; LC3-Ⅱ, microtubule-associated protein 1A/1B-light chain 3-Ⅱ; mTORC1, mechanistic target of rapamycin complex 1; ROS, reactive oxygen species.

### Current limitations

Despite emerging insights into the autophagy-ferroptosis interplay in PF, the translation of these findings into clinical practice remains hindered by several unresolved challenges. First, current research predominantly relies on bleomycin-induced PF animal models, which simulate acute post-injury fibrogenesis but fail to recapitulate the spatiotemporal heterogeneity of human PF (*e.g.*, subpleural fibrosis distribution and temporal progression from injury to chronic remodeling in IPF). These models lack cell-type-specific dynamics, as bleomycin-driven acute inflammation contrasts with human IPF's chronic, multicellular cascades involving spatially distinct interactions among epithelial cells, fibroblasts, and immune populations within fibrotic niches. Second, the crosstalk between autophagy and ferroptosis in orchestrating core PF events (*e.g.*, fibroblast hyperactivation in fibroblastic foci, spatially restricted EMT and ECM remodeling) remains insufficiently characterized. Crucially, their cell-type-specific roles (*e.g.*, ferroptosis susceptibility in alveolar epithelial cells *vs.* autophagy dysregulation in fibroblasts) and spatiotemporal dynamics (*e.g.*, early injury response *vs.* chronic fibrotic maintenance) in human PF are poorly defined. While autophagy may mitigate ferroptosis by clearing damaged mitochondria and lipid peroxidation products, its synergistic or antagonistic interplay across distinct cellular compartments and disease phases lacks human validation. Third, existing therapies targeting isolated nodes (*e.g.*, rapamycin or liproxstatin-1) show limited efficacy because of compensatory pathways and PF's multifactorial nature, further compounded by limited understanding of cell-type-specific responses in human microenvironments and temporal adaptation of pathogenic cells. Finally, the absence of spatiotemporally resolved methodologies for human tissues (*e.g.*, dynamic imaging of organelle interactions in specific cell types and spatial multi-omics mapping of fibrotic niches across PF stages) hinders mechanistic elucidation. Bridging this gap requires advanced tools such as single-cell spatial transcriptomics and real-time tracking to decode human PF's cellular and spatiotemporal complexity. For example, single-cell RNA sequencing characterizes transcriptomes at cellular resolution, elucidating molecular mechanisms underlying pathogenesis in PF. Multiple studies have demonstrated the heterogeneity and cell-cell interactions in the progression of PF. For example, a recent study found that the crosstalk between C-X-C motif chemokine ligand 10 (CXCL10)^+^/matrix metallopeptidase 14 (MMP-14)^+^ monocytes and C-C motif chemokine ligand 3 (CCL3)^+^ neutrophils promotes silica-induced PF based on single-cell RNA sequencing^[144]^. Furthermore, Adams *et al*^[145]^ revealed the complexity and diversity of aberrant cellular populations in IPF, including aberrant basaloid cells and profibrotic macrophage populations, by using single-cell RNA sequencing. Spatial transcriptomics enables comprehensive characterization of cellular heterogeneity and molecular pathobiology within the adult distal lung across healthy and pulmonary fibrotic states. Spatial transcriptomics may facilitate the identification of conserved, molecularly defined spatial niches, and their disease-associated evolution to provide mechanistic insights into PF pathogenesis^[146]^.

## Conclusions

Autophagy and ferroptosis, each characterized by distinct molecular mechanisms and morphological features, engage in a complex interplay during PF pathogenesis. This interplay involves critical determinants such as redox imbalance, iron dysregulation, and various signaling cascades. A comprehensive understanding of these interactions is essential for elucidating the pathogenesis of PF and developing innovative therapeutic strategies. To accelerate translational progress, future investigations should prioritize the following approaches: (1) Single-cell multi-omics approaches integrating various other omics techniques, including epigenomics, transcriptomics, and proteomics, to map autophagy-ferroptosis crosstalk across heterogeneous cell types in human PF niches; (2) *In vivo* lineage tracing models integrating fluorescence reporters to dynamically track cell fate transitions (*e.g.*, EMT, FMT, and EndoMT) regulated by this interplay; and (3) Mechanistically driven clinical trials to test cell-type-targeted therapies. Moreover, it is vital to effectively integrate emerging technologies with clinical practice and develop novel targeted therapies to achieve precision treatment. Most importantly, conclusions drawn solely from human IPF-sample evidence are explicitly restricted to IPF and should not be broadly generalized to all forms of PF.

Although previous reviews have addressed the roles and therapeutic implications of ferroptosis and autophagy in PF, this review focuses specifically on synthesizing the available evidence for their bidirectional crosstalk and its potential contribution to PF progression. This crosstalk represents an emerging mechanistic framework that may regulate PF pathogenesis, thereby broadening the mechanistic perspective beyond ferroptosis or autophagy alone. Accordingly, our synthesis highlights how this integrated view could inform novel therapeutic strategies and identifies potential drug targets that merit further investigation in PF treatment.
